# Highly-Sensitive Allele-Specific PCR Testing Identifies a Greater Prevalence of Transmitted HIV Drug Resistance in Japan

**DOI:** 10.1371/journal.pone.0083150

**Published:** 2013-12-16

**Authors:** Masako Nishizawa, Junko Hattori, Teiichiro Shiino, Tetsuro Matano, Walid Heneine, Jeffrey A. Johnson, Wataru Sugiura

**Affiliations:** 1 AIDS Research Center, National Institute of Infectious Diseases (NIID), Tokyo, Japan; 2 Clinical Research Center, National Hospital Organization Nagoya Medical Center, Nagoya, Japan; 3 Division of HIV/AIDS Prevention, Centers for Disease Control and Prevention, Atlanta, Georgia, United States of America; 4 Department of AIDS Research, Nagoya University Graduate School of Medicine, Nagoya, Japan; INSERM, France

## Abstract

**Background:**

The transmission of drug-resistant HIV in newly identified infected populations has become an underlying epidemic which can be better assessed with sensitive resistance testing. Since minority drug resistant variants cannot be detected by bulk sequencing, methods with improved sensitivity are required. Thus, the goal of this study was to evaluate if transmitted drug resistance mutations at minority levels in Japanese patients could be identified using highly sensitive allele-specific PCR (AS-PCR).

**Materials and Methods:**

Samples were taken from newly diagnosed HIV/AIDS cases at the National Nagoya Hospital from January 2008 to December 2009. All samples were bulk sequenced for HIV protease and reverse transcriptase. To detect minority populations with drug resistance, we used AS-PCR with mutation-specific primers designed for seven reverse transcriptase inhibitor resistance mutations, M41L, K65R, K70R, K103N, Y181C, M184V, and T215F/Y, and for three protease inhibitor resistance mutations, M46I/L and L90M.

**Results:**

We studied 149 newly identified HIV cases. Bulk sequencing detected 8 cases with NRTI resistance mutations (one with A62V, one D67E, one T215D, one T215E, two with T215L and two T215S) and 15 with PI resistance mutations (one with N88D and 14 with M46I). Results obtained by AS-PCR and bulk sequencing demonstrated good concordance but the AS-PCR enabled the detection of seven additional drug-resistant cases (one M41L, two with K65R, two with K70R, and one M184V) in the RT region. Additionally, AS-PCR assays identified 15 additional cases with M46I, five with M46L and four cases with L90M in the protease region.

**Conclusions:**

Using AS-PCR substantially increased the detection of transmitted drug resistance in this population from 15.4% to 26.8%, further supporting the benefit of sensitive testing among drug-naïve populations. Since the clinical impact of minority drug-resistant populations is not fully comprehended for all mutations, follow-up studies are needed to understand their significance for treatment.

## Introduction

The use of combination antiretroviral therapy of (cART) has resulted in sustained reductions in morbidity and mortality from HIV infection [1,2]. Five classes of antiretrovirals (ARVs) are currently available in clinical use in Japan. However, selection of drug resistance mutations during cART is still a major issue affecting the clinical efficacy of ARVs and prognosis of HIV infected individuals [3,4]. 

A United States Department of Health and Human Services (DHHS) guideline recommends drug resistance testing for patients before they begin cART to guide their therapy[5]. Conventional bulk sequencing is used to detect drug resistance mutations in viral RNA from patient plasma, but the method generally does not detect mutants that comprise less than 20% of the viral population in individuals [4-7]. This detection limitation is a concern, both because transmitted minority variants might persist at low frequencies and most newly diagnosed HIV infections are in persons who have been infected for several months to years, providing time for drug resistant viruses with reduced viral fitness to decay to levels that conventional testing is not able to detect [8-10]. Therefore, the ability to detect low-frequency variants below 20% would improve identification of infections involving drug-resistant viruses and better inform decisions on the selection of active ARVs, especially for persons initiating treatment with NNRTI regimens. To detect low-frequency variants, several methods were developed and used to analyze drug-naïve persons and drug-experienced persons [11-14]. Several studies have shown the advantages of highly sensitive drug resistance assays with women who received intrapartum single-dose nevirapine (SD-NVP) for the prevention of mother-to-child HIV transmission. These reports on testing for NVP resistance have found that drug resistance emerges more frequently and persists for longer than previously demonstrated by bulk-sequencing. Persisting minority NVP-resistant viruses may result in poor virologic responses when subsequent regimens contain nevirapine-related drugs [15-19].. We previously reported that highly-sensitive drug resistance testing that is based on allele-specific real-time PCR can detect minority drug-resistant variants both in infections reported to be wildtype and infections involving other resistance mutations as determined by bulk sequencing. As with majority-level resistance, the amount of low-frequency resistance in new infections reflects both the prevalence of cART use in the region and behavior that is inconsistent with prevention practices for persons on therapy [20]. 

Recently, it has been reported that the prevalence of drug-resistant HIV transmission among newly diagnosed patients analyzed by bulk sequencing is increasing in Japan, rising from 5.9% in 2003 to 8.3% in 2008 [21]. As the study concluded, this observation was seen not only for recently infected persons, but also chronically infected but recently diagnosed cases, raising concern over the amount of resistance detection lost due to reversion. Therefore, by use of a highly sensitive method in the current study we attempted to examine for the possibility and prevalence of transmitted drug resistant mutations hidden as minority populations.

## Materials and Methods

### Ethics statement

Specimens were anonymous residual diagnostic material from subjects who provided written consent for HIV testing. The Ethical Committee for Biomedical Science of the National Institute of Infectious Diseases determined that this testing did not involve identifiable human subjects and has approved the study.

### Samples

**Figure 1:**
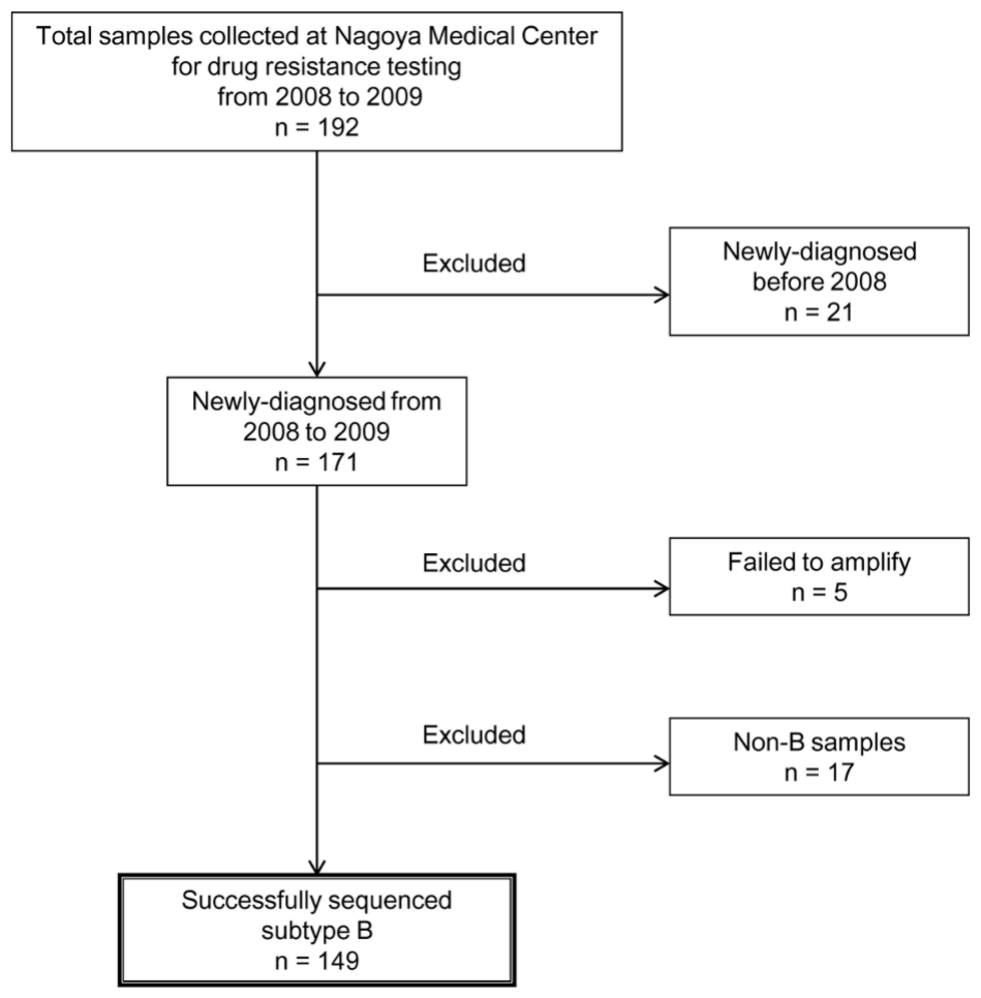
Flow Diagram of sample selection. Flow diagram of sample selection for analysis of minority drug resistance mutations in the HIV-infected patient samples newly diagnosed at National Nagoya Hospital from 2008 to 2009.

The 192 plasma samples were collected from HIV/AIDS cases for drug resistance analysis from January 2008 to December 2009 in National Nagoya Hospital (Table 1). Among these, 149 cases of newly diagnosed HIV-1 subtype B-infected ART-naïve individuals were selected and analyzed in this study (Figure 1). All samples were collected as part of HIV surveillance studies under Institutional Review Board of National Institute of Infectious Diseases, and written informed consent was obtained from each patient. These samples were directly sequenced for HIV protease (PR) positions 1-99 and reverse transcriptase (RT) positions 1-240. Drug resistance mutations were defined according to the mutation list proposed by Bennett et al. 2009[22]. All testing was performed by the NIID AIDS Research Center in Tokyo, Japan[21].

**Table 1:** Demographics of samples.

		2008	2009	Total
**Total**		75	74	149
**Gender**	male	74	73	147
	female	1	1	2
	unknown	0	0	
**Age**	median(Q1, Q3)	39	38	39
**Risk behavior**	MSM	52	49	101
	Sexual	9	11	20
	MSM/Sexual	8	13	21
	Hemophiliac	1	0	1
	Unknown	5	1	6
**VL**	Median	9.70E+04	7.00E+04	7.90.E+04
	mode	1.10E+05	2.70E+04	1.10.E+05
**CD4**	average	199.7	225.4	212.0

### RNA extraction and virus template amplification

HIV RNA was extracted by Roche High Pure Viral RNA Kit from 200uL plasma samples. RNA was reconstituted in 100 uL of DEPC water and stored at -80°C until use. The HIV protease-reverse transcriptase (PR-RT) region was amplified by one-step RT-PCR (TAKARA One Step RNA PCR kit) with forward primer (DRPRO5 : AGA CAG GYT AAT TTT TTA GGG A) and reverse primer (DRRT34 : GCT ATT AAG TCT TTT GAT GGG TCA TA). RT-PCR amplification conditions were 55°C for 40 minutes and 40 cycles of 95°C for 10 seconds, 52°C for 5 seconds, and 72°C for 90 seconds. In the case that the amplification of RT- PCR did not generate sufficient template, nested-PCR was performed using forward primer (PROFWD1F : CAG ATC ACT CTT TGG CAA CGA CC) and reverse primer (GEN4R : ATC CCT GGG TAA ATC TGA CTT GC)[23]. Nested-PCR amplification condition was 94°C 1 minute and 30 Cycles of 94°C for 10 seconds, 55°C for 4 seconds and 74°C for 15 seconds. 

### Real-time PCR (AS-PCR)

To detect minority populations with drug resistance, we used highly sensitive allele-specific PCR validated for subtype B HIV as described [17,23]. Briefly, mutation-specific primers were designed for seven reverse transcriptase inhibitor resistance mutations, M41L, K65R, K70R, K103N, Y181C, M184V, and T215F/Y. Results of highly sensitive allele-specific PCR and population sequencing data were compared for concordance and presence of additional mutations. The HIV-1 total copy primers, Com2F and Com4BR, span n.t. 258–420 in RT and were used with the common probes, Com1P and 2P (Table S1)[17,23]. For multiple mutation screening, several resistance mutation-specific reactions can be performed simultaneously. The cycle number at which the fluorescence emission exceeds the background fluorescence threshold is the threshold cycle (CT) and is the unit of measure for comparing the differences in amplification signals (∆CT) between the total copy and mutation-specific reactions. All samples were tested in duplicate with the means of the total copy and mutation-specific CTs used for the determination of the ∆CT. Each ∆CT cutoff value for interpreting the presence of drug resistance mutations was determined previously [23] and were between 8.5 from 10.5 cycles, for validated assay cut-offs ranging from 0.03% to 2.0% mutant, depending on the assay. 

Real-time PCRs were initiated with a hot-start incubation at 94°C for 11 minutes before proceeding to 45 cycles of melting at 94°C for 30 seconds, annealing at 50°C for 15 seconds and extension at 60°C for 30 seconds. All reactions were performed in a total volume of 50 uL/well in 96-well PCR plates using iQ5 real-time PCR thermocyclers with optical units (Bio-Rad) and AmpliTaq Gold polymerase (2.5 U/reaction; Applied Biosystems). Final reagent concentrations were 320 nM for the forward and reverse primers, 160 nM probe(s), and 400 mM dNTPs.

### M46I/L primers for real-time-PCR

For this study, new primers for the detection of M46I and M46L protease inhibitor mutations were constructed as described before [17,23,24]. As with the RT primers, the protease mutation-specific primers (Table S2) were designed to preferentially anneal with the targeted mutation nucleotide(s), thus having reduced affinity for wild-type sequences. Specificity was enhanced by creating designed mismatches at the -2 nucleotide position relative to the primer 3’-end for each primer. Furthermore, to compensate for the spectrum of polymorphisms present, mixtures of three uniquely designed forward primers were required to detect M46L. Mutation-specific primer mixtures were experimentally evaluated and the ratios that best balanced differences in primer avidities and minimized cross-interference in primer annealing were selected. 

### Site-directed mutagenesis and cloning

M46I and M46L mutant clones for plasmid development were constructed by site-directed mutagenesis using HXB2 as a template. These constructs were used as positive control to verify the M46I/L primers and probes. To insert M46I or M46L mutations into HXB2, PCR with KODplus was performed using a pair of complementary primers (M46I-forward primer : GAA GAT GGA AAC CAA AAA TaA TAG GGG GAA TTc GAG G, M46L(ttg)-forward primer : GAA GAT GGA AAC CAA AAt TGA TAG GGG GAA TTG GAG G , M46L(ctg)-forward primer : GAA GAT GGA AAC CAA AAc TGA TAG GGG GAA TTG GAG G), and reverse primer : CTG GCA AAC TCA TTT CTT CTA ATA CTG TAT CAT CTG CTC C). PCR amplification conditions were 94°C for 2 minutes and 35 cycles of 98°C for 10 seconds, 68°C for 2 minutes and 30 seconds.

### Evaluation of the new protease assays on plasmids and clinical samples

HXB2-M46I, HXB2-M46L and HXB2 (wild-type) plasmids were used in the preliminary selection of primer mixtures that provided the greatest sensitivity and specificity. The absolute mutation detection limits for the primer mixtures, that is, the greatest ΔCT that was able to distinguish mutant viruses from wild-type, were estimated from triplicate testing of mutant clone serial dilutions. The assays evaluated mutation-containing sequences at frequencies between 100%-0.0001% in a wild-type background, with each dilution having same total plasmid copies. The ΔCTs generated from the mutant dilutions were compared to the ΔCTs generated with the wild-type plasmids alone. Solely for the purpose of comparing relative assay detection limits with finite virus sequences, the ΔCT within the linear dilution range (R^2^>0.995) that was equivalent to a frequency increase of 0.5 log_10_ above the wild-type mean ∆CT was chosen as the absolute assay detection limit. Selecting the detection limit in this manner provided an adequate buffer against variability in wild-type sequences and also took into account the PCR efficiency of the assay (slope of the dilution curve).

 To evaluate cutoff values of M46I and M46L in patient samples, PR-RT sequences derived from ART-naïve patients were analyzed by real-time PCR. Forty-two PR-RT region sequences derived from 16 patients were cloned by TA-cloning to serve as heterogeneous wild-type sequences. Fifty-five samples with protease M46I were obtained from 20 individuals and 22 samples with M46L were obtained from 12 individuals. To increase the stringency of assay evaluations, specimens with substantial numbers of polymorphisms in primer binding sites were also included.

### Assessing mutation associations in mutation-specific amplicons

To evaluate whether additional information on resistance mutations could be gained from the real-time PCR assays, we performed bulk sequencing (BigDye reagent, Prism 3130xl analyzer, Applied Biosystems) of the products from M46I/L or L90M-specific reactions to assess mutation linkage. Mutation-specific amplicon sequences were compared to their respective sample bulk sequence for evidence of nucleotide differences. Any other resistance mutation(s) found in the mutation-specific amplicons would indicate that they were on the same viral strand(s) as the mutation that was specifically targeted by the primers. 

### Phylogenetic analysis

Protease sequences were aligned by means of the clustal-W program with a set of reference sequences recommended by the Los Alamos sequence database (http://www.hiv.lanl.gov/content/index). The results of the alignment were then analyzed by the neighbor-joining method using MEGA5 [24,25]. In order to analyze the relationship between M46I/L-positive amplicon sequences and bulk sequences, we extended the M46I/L-positive amplicon by using the PRO2L reverse primer (Table S1B), which allowed sequencing from PR codon 47 to RT codon 36 of these M46I/L-positive amplicons (270 bp). In the case of L90M amplicon analysis, 209 bp DNA fragments extending from amino acid 20 in PR to amino acid 89 in PR were represented in the phylogenetic tree. The phylogenetic relatedness of these mutation-containing amplicons excluding the resistance codon position were represented in trees constructed using Kimura 2-parameter model with a discrete gamma distribution [1 +G] and 500 bootstrap replications in MEGA5.

### Statistics

The Mann-Whitney U test was used to test for differences in CD4 counts and VL between the groups with minority drug resistance mutations and those without minority drug resistance mutations. 

## Results

### Minority M46I could be detected as low as 0.04% and M46L could be detected as low as 0.03% in site-directed mutant clones

**Figure 2:**
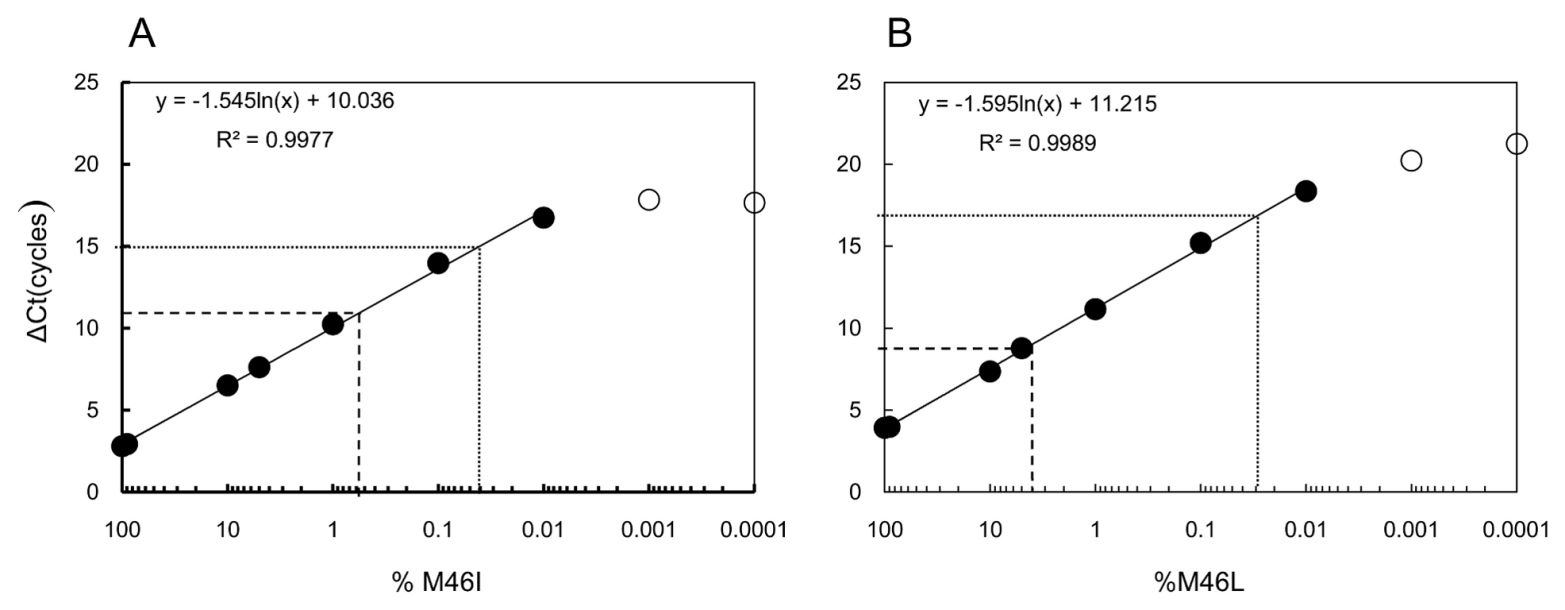
Mutation-specific assay reactivity on plasmids. Cloned M46I (A.) and M46L (B.) mutant virus template was diluted 10-fold, from 100% to 0.0001%, in backgrounds of wild-type sequence to determine assay detection limits. Plotted are the mean ΔCT versus log_10_ of the mutant dilution series. The lower detection limit (lower dotted line) was placed at the ΔCT equivalent to 0.5 log_10_ below (0.5-log greater reactivity than) the wild-type ΔCT. For comparison, the mutant virus frequency equivalences for the established clinical cutoffs are also shown (dashed line).

Relative limits of detection were compared in a simple laboratory setting using serial dilutions of HXB2-M46I or HXB2-M46L in backgrounds of HXB2 wildtype plasmid. The ΔCT that was equivalent to a 0.5 log greater reactivity than the wild-type mean ΔCT on the dilution curve (M46I : ∆CT=15 cycles, M46L : ∆CT=17 cycles) was used to compare assay sensitivities (Figure 2). This approach yielded detection limits of 0.04% and 0.03% for M46I and M46L, respectively. As this was derived from cloned sequences this is a theoretical detection limit against which clinical specimens are evaluated.

### High sensitivity and specificity of M46I/L detection assays confirmed with clinical samples

**Figure 3:**
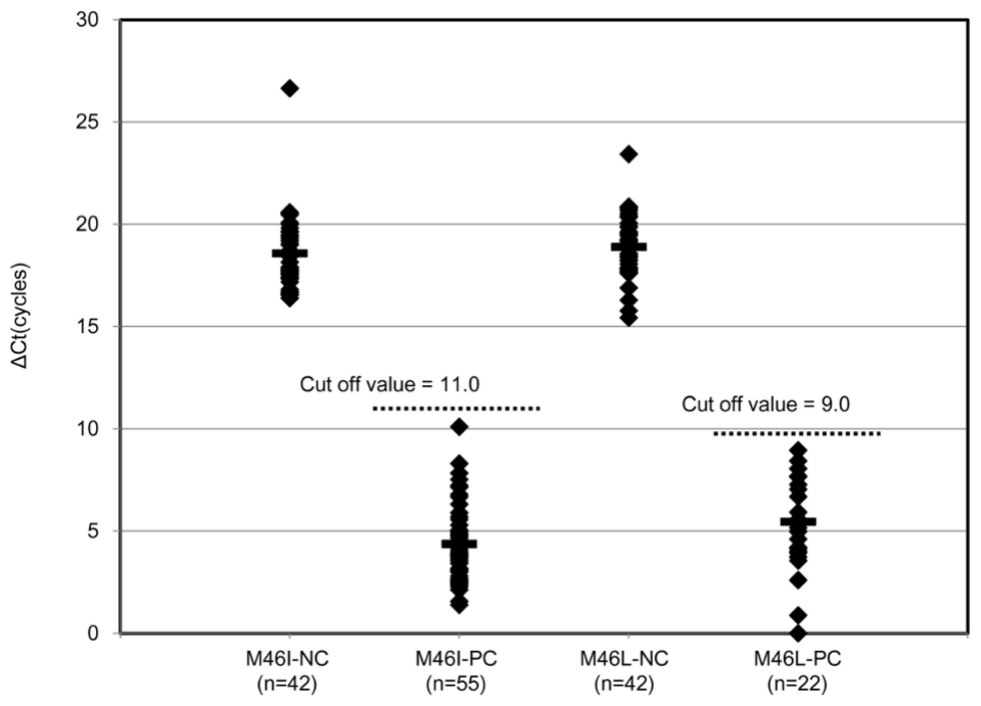
Assay reactivity with wild-type and M46I/L mutation clinical samples. Dotted ΔCT values from clinical samples with sequence-detectable mutations and with wild-type sequences are shown. The range of reactivity for each assay is shown for wild-type and mutant samples. The mean of ΔCT (bar) for each group is indicated. Assay cutoffs (dotted horizontal line) were established to exclude all wild-type viruses from detection. PC; Positive clones with M46I/L, NC; Negative clone with no M46I/L(wild type).

Assay cutoff values intended for population-wide clinical screening were established using 42 cloned wild-type sequences derived from 14 patient-derived specimens collected by NIID from 2005-2007. The assay cutoffs selected based on plasmid sequences were evaluated against clinical specimens using a total of 55 samples with sequence-detectable M46I mutation and a total 22 samples with sequence-detectable M46L mutation. The resulting distribution of collated ΔCTs from the wild-type samples supported a ΔCT cutoff of 11 cycles for M46I clinical testing (ΔCTs ranged from 16.39–26.65 cycles) (Figure 3 and Table S3). Extrapolating from the dilution curve for cloned M46I sequences, this cycle difference corresponded to a frequency mean of 0.54% mutant virus (see Table S3). At this cutoff, all 55 genotyped M46I samples were positive (ΔCTs ranged from 1.39 to 10.1 cycles, Figure 3 and Table S3). For M46L assay, ΔCT cutoff was 9 cycles to avoid low-level amplification from spurious primer binding against clinical quasispecies specimens; this cutoff placement corresponded to a frequency mean of 4.01% mutant virus. All genotype M46L samples were positive (ΔCT ranged from 0.88 to 8.95 cycles) (Figure 3 and Table S3). Because of unusual polymorphisms, some samples comprised almost entirely of mutant virus produced ΔCTs near the cutoff. In these situations, elevated ΔCTs resulting from weak primer binding could be interpreted as mutant viruses present at low frequencies. Hence, this testing format is best-suited to provide highly specific population-level resistance screening and is not necessarily applicable to mutant virus quantitation.

### AS-PCR method identifies a greater prevalence of transmitted HIV drug resistance

Samples from a total of 149 subtype B cases were collected at Nagoya Medical Center for drug resistance testing. Drug resistance mutations were initially analyzed by bulk sequencing, and 23 cases were found to possess drug resistance mutations. As summarized in Table 2, all resistant mutations were found as sole mutation, one with A62V, one with D67E and six cases of intermediates at codon 215 (one with T215D, one with T215E, two cases of T215L and two cases of T215S), one with N88D and 14 cases of M46I mutation were detected by conventional bulk sequencing analysis, yielding a drug resistance mutation prevalence of 15.4% (23/149 cases) (Table 2). The sensitive screening detected an additional one case of M41L (0.67%), two cases of K65R (1.34%), two cases of K70R (1.34%), one case of M184V (0.67%), 15 cases of M46I (19.46%), 5 cases of M46L (3.36%) and 4 cases of L90M (2.68%) as minority-level drug resistance mutations (Table 2). The identified A62V, D67E, T215E, T215S and N88D mutations detected by bulk sequencing were not targeted by AS-PCR, and therefore were not included in determining changes in mutation frequency. All 17 mutations detected by bulk sequencing analysis that were also targeted by AS-PCR were likewise detected by the sensitive PCR method. The combined prevalence of drug resistance mutations in the total of 149 cases was 26.8% (40/149 cases) (Table 2). In one case, six mutations, M41L, K70R, M184V, M46I, M46L and L90M were detected as minority mutations by the highly sensitive assays (ID 29). These six mutations were undetectable by bulk sequencing analysis. In other cases, K70R and M46I were detected (ID 22), and M46I and M46L were detected in another case as minority drug resistance mutations (ID 5). Of those with minority drug resistance mutations, 11 cases were from 2008 and 12 cases were from 2009. The majority of patients with minority variants were MSM (90.9% in 2008 and 83.3% in 2009) and Japanese, and no significant differences were observed in viral load and CD4 counts by Mann-Whitney U test (*p*=0.17 and *p*=0.308, respectively) for persons with or without mutations. Though all of the samples from 2008 were Japanese patients, three cases from 2009 were non-Japanese patients (Table 2). 

**Table 2:** Characteristics of HIV/AIDS patients with drug resistance mutations.

							Bulk-seq		AS-PCR	
ID	Gender	Risk behavior	Year	Nationality	VL	CD4	RT mutations	PR mutations	RT mutations	PR mutations
1	M	MSM	2008	Japan	2.0.E+04	402	A62A/V			
2	M	Heterosexual	2008	Japan	1.2.E+06	14		N88D/N		
3	M	Heterosexual	2008	Japan	3.1.E+05	38	T215L		T215F*	
4	M	MSM/Heterosexual	2008	Japan	5.8.E+05	222	D67D/E			M46I
5	M	MSM	2008	Japan	2.6.E+04	481				M46I, M46L
6	M	MSM	2008	Japan	1.7.E+06	10				M46I
7	M	MSM/Heterosexual	2008	Japan	4.1.E+05	39	T215S			
8	M	MSM	2008	Japan	2.2.E+05	14		M46I		M46I
9	M	MSM	2008	Japan	1.5.E+04	356				L90M
10	M	MSM/Heterosexual	2008	Japan	1.2.E+05	28	T215S			
11	M	MSM/Heterosexual	2008	Japan	1.3.E+05	348		M46I		M46I
12	M	MSM	2008	Japan	2.1.E+04	391			K65R	
13	M	MSM	2008	Japan	2.1.E+05	553		M46I		M46I, L90M
14	M	Heterosexual	2008	Japan	6.7.E+04	153		M46I		M46I
15	M	MSM	2008	Japan	2.2.E+05	10		M46I		M46I, M46L
16	M	MSM	2008	Japan	1.2.E+03	45	T215L		T215F*	M46I
17	M	MSM	2008	Japan	2.6.E+04	750		M46I		M46I
18	M	MSM	2008	Japan	1.1.E+05	146		M46I		M46I
19	M	MSM	2008	Japan	8.4.E+04	11				M46I
20	M	MSM	2008	Japan	1.4.E+05	86		M46I		M46I
21	M	MSM	2008	Japan	1.1.E+05	1050				M46I
22	M	MSM	2008	Japan	2.2.E+04	154			K70R	M46I
23	M	MSM	2009	Japan	7.2.E+03	319				M46L
24	M	MSM	2009	Japan	2.9.E+04	185				M46I
25	M	Heterosexual	2009	Japan	1.6.E+04	290	T215D		T215Y**	
26	M	MSM/Heterosexual	2009	Japan	2.7.E+04	442		M46I		M46I
27	M	MSM	2009	Brazil	8.6.E+04	14		M46I		M46I, M46L
28	M	MSM	2009	Argentina	2.0.E+05	28				M46I
29	M	MSM	2009	Japan	4.5.E+05	3			M41L, K70R, M184V	M46I, LM46L, L90M
30	M	MSM	2009	Japan	1.7.E+05	nt		M46I		M46I
31	M	MSM	2009	Japan	2.9.E+05	32	T215E		T215Y	
32	M	MSM	2009	Japan	1.1.E+05	833		M46I		M46I
33	M	MSM	2009	Japan	4.1.E+04	426		M46I		M46I
34	M	MSM	2009	Japan	1.2.E+07	441		M46I		M46I
35	M	MSM	2009	Australia	1.5.E+04	1				M46I
36	M	MSM	2009	Japan	2.2.E+04	324				L90M
37	M	MSM	2009	Japan	6.0.E+04	373				M46I
38	M	MSM	2009	Japan	2.9.E+04	nt			K65R	
39	M	Heterosexual	2009	Japan	4.5.E+03	974				M46I
40	F	Heterosexual	2009	Japan	8.0.E+04	nt				M46I
41	M	MSM	2009	Japan	1.4.E+04	300				M46I
Subtotal							8(5.3%)	15(10.1%)	8(5.3%)	32(21.5%)
Total							23/149(15.4%)		40/149(26.8%)	

* T215F detection primers can detect T215L, T215I and T215V. **T215Y detection primers can detect T215D, T215H and T215N.

### Sequence analysis of M46I and M46L-specific amplicons showed that these mutations were not linked to L90M in the patient samples

To analyze the linkage between drug resistance mutations, we directly sequenced positive M46I/L or L90M-specific PCR products to ascertain whether additional genotypic information could be obtained from those amplicons. In ID 29, the I72V polymorphism observed in the bulk sequence was detected in M46L and L90M amplicons, but not in the M46I amplicon (Table 3). Additionally, M46I/L mutations were not detected in the minority L90M amplicon indicating these mutations were not linked. In ID 27, A71T was detected in the M46L amplicon, but this mutation was not found in the M46I amplicon or the bulk sequence (Table 3). In ID 22, the M46I amplicon matched the bulk sequence.

**Table 3:** Genetic linkage of M46I/L or L90M and other mutations.

Sample	Sequences	Mutations
ID 29	Direct-sequencing	I62V, L63P, *I72V*, T74A, V77I, I93L
	M46I amplicon*	**M46I**, I62V, L63P, T74A, V77I
	M46L amplicon*	**M46L**, I62V, L63P, *I72V*, T74A, V77I
	L90M amplicon**	I62V, L63P, *I72V*, T74A, V77I, **L90M**
ID 27	Direct-sequencing	**M46I**, E21R, R41K, I62V, L63P, L89I, Q92K, I93L
	M46I amplicon*	**M46I**, I62V, L63P
	M46L amplicon*	**M46L**, I62V, L63P, *A71T*
ID 22	Direct-sequencing	E35D, M36I, L63P, H69K, V77I
	M46I amplicon*	**M46I**, L63P, H69K, V77I

^*^ M46I and M46L amplicons were spanned from M46 to N88.

^**^ L90M amplicon was spanned from I15 to L90.

### Phylogenetic relatedness of minority and bulk sequence-detectable resistance mutations

**Figure 4:**
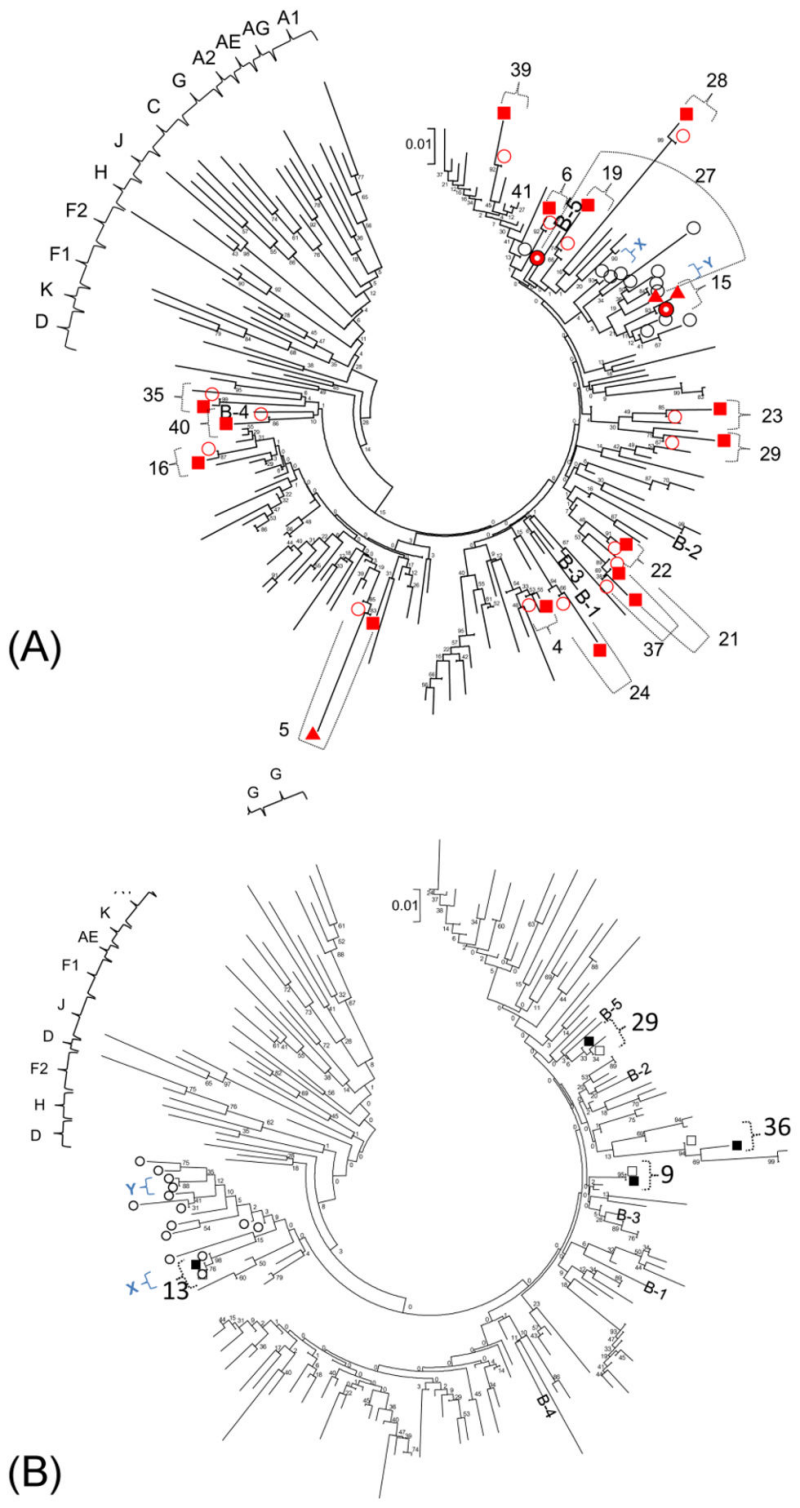
Phylogenetic tree of samples with or without minority variants of M46I/L or L90M drug resistance. A neighbor-joining phylogenetic tree of all protease sequences using the Kimura 2-parameter model was generated in MEGA5. Numbers shown are IDs of patients with detectable minority drug resistance. Open circles (black color) are virus with M46I detected by bulk sequencing. A. Solid squares (red color) indicate sequences of M46I-specific amplicons and solid triangle (red color) indicate sequences of M46L amplicons; open circles (red color) indicate bulk sequences for persons with minority M46I/L mutations; X and Y are pairs of closely related transmitted M46I sequences. B. Solid squares indicate L90M-specific amplicon sequences; open squares indicate bulk sequences for persons with minority L90M mutations. Open circles (black color) are virus with M46I detected by bulk sequencing. Abbreviations of subtype B references: B-1; B.NL.00.671 00T36.AY423387, B-2; B.US.98.1058 11.AY331295, B-3; B.FR.83.HXB2 LAI IIIB BRU.K03455, B-4; B.TH.90.BK132.AY173951, B-5; B.US.98.15384 1.DQ853463.

Phylogenetic analysis conducted on all the protease sequences produced a pattern consistent with good separation of unrelated sequences even though they were not supported at the roots by significant bootstrap values due to somewhat short sequence lengths (Figure 4A). However, the branch tips show strong bootstrap support for the relatedness of minority variants to the patient bulk sequences from which they were derived. Moreover, some of the patients with bulk sequence-detectable M46I appeared to group together with relatively high bootstrap values (pairs X and Y, Figure 4A) and may represent infections linked within transmission clusters. The sequences within each X and Y pair were 100% identical in the 270 bp analyzed, with the exception of ≤3 mixed-base positions that included the nucleotide of the paired patient. In assessing the relatedness of the four detected minority L90M to infections that have the PR M46I mutation, three L90M were from patients that were wildtype at codon 46, the fourth was ID 13 which was also an M46I case (Figure 4B). 

## Discussion

In this study, we used a highly sensitive method to screen for minority drug-resistant populations in 149 cases of newly diagnosed HIV-infected patients. An additional six drug resistance mutations in the RT region and thirty drug resistance mutations in PR region were detected as minority-level drug resistance mutations. For the ten codons associated with resistance (RTI mutations: M41L, K65R, K70R, K103N, Y181C, M184V and T215F/Y, PI mutations: M46I/L and L90M) the prevalence of detectable drug resistance mutations increased from 15.4% to 26.8% using the highly sensitive assays. A previous surveillance study in Japan using bulk sequencing reported that in 2008 the prevalence of transmitted drug resistance was 8.3% [21]. Therefore, drug resistance mutation surveillance analyzed by bulk sequencing underestimates transmitted drug resistance, which potentially has both clinical and epidemiologic implications.

The epidemiologic implications of increased transmitted resistance may reflect prevention failures in persons who know they are infected and transmit HIV to their partners. The clinical implications of minority drug resistance center around the impact of these viruses on ART responses. Previous studies have reported that minority NNRTI-resistant variants are associated with increased risk of virologic failure in patients receiving first-line NNRTI-based ART regimens [20,26-32]. These findings are important because NNRTI resistance is the most commonly transmitted resistance in the US and Europe [33,34]. However, no evidence of either majority or minority NNRTI-resistance was found in this study, a unique finding that is explained by the historically infrequent use of NNRTIs in ART regimens in Japan. Instead, we note that the NRTI K65R and M184V mutations were both detected as minority populations. As major mutations K65R and M184V reduce the clinical efficacy of TDF and 3TC/FTC, respectively [35-37], however their clinical impact as minority mutations is not fully clear. 

A previous study demonstrated no impact on therapy responses in patients who had minority-level K65R and M184V mutations when provided regimens that included protease inhibitors; however; because of the small number of patients representing different treatment regimens in that study, the bearing of these mutations could not be fully evaluated [29]. Additional studies are needed to assess clinically significant frequencies of different NRTI-resistant variants on various treatment regimens. In the present study, one of the 149 cases evaluated possessed six drug resistance mutations as minority variants (Table 2). Genotype interpretation by the Stanford HIV Drug Resistance Database showed that the patient possessed high-level resistance to NRTIs and some PIs. It was not possible to follow the clinical course of this patient to elucidate the significance of minority variants on subsequent cART.A major finding in this study was the high prevalence of transmitted PI resistance (20%) which accounted for about two-thirds the overall transmitted resistance. The high prevalence of transmitted PI resistance is supported both by detection at majority as well as minority variant levels, the latter comprising more than half of the transmitted PI cases. The high prevalence of PI resistance can be explained by the longstanding and predominant use of PI-based regimens in Japan, including darunavir and atazanavir in both first-line and second-line regimens 

Genetic linkage analysis provided more insights into the composition of the viral population by showing that L90M, M46I and M46L in many patients existed on separate viral genomes. The capacity to identify linked mutations could be important for understanding the persistence [38] and clinical impact of mutant variants. A major factor that influences the persistence of drug-resistant mutants *in vivo* is their relative replicative capacity within the viral population. *In vitro* competition experiments conducted in the absence of drugs have shown that drug resistance mutations impair replicative fitness by different degrees. For instance, the M46I, the K70R, the 215 intermediate mutations have a lesser impact on fitness than L90M, K65R, and M184V [38-40], and, thus, such mutations are likely to persist longer *in vivo*. Moreover, accumulation of compensatory mutations such as L63P and A71V in protease have been demonstrated to increase or restore replicative fitness of PI resistant variants, and that once compensation has taken place reversion to wildtype is prohibited by a less fit intermediate [41]. This may explain, for example, the high prevalence of M46I we detected in this study as bulk and minority species. 

Phylogenetic analysis showed the sequences of minority M46I/L and L90M-positive amplicons were closely related to their source bulk sequences, supporting that the minority sequences detected were unique to the respective patients and were not the result of contamination. A few cases possessing the M46I mutation by bulk sequencing demonstrated a strongly supported identity which was not biased by including the resistance mutations in the analysis. However, with regard to minority M46I variants, we found they did not cluster closely with viruses from persons with majority-level M46I. This suggested that at least two pairs with majority-level M46I were phylogenetically related whereas those with minority M46I were scattered among the transmitted virus population, unrelated to any of the other cases in our analysis. While the sequence lengths used in the analysis might limit our ability to draw robust bootstrap values deeper in the tree nodes, the sequences for the pairs within the two clusters were identical with the exception of a few positions with mixed bases, and were further supported by high bootstraps. 

The ability to conduct surveillance of minority-level drug resistance mutations is an important advancement to help understand transmission of HIV drug resistance in Japan. The finding from our select analysis of mutations of interest cannot be extrapolated to all codons associated with drug resistance; however, these results suggest that a substantial proportion of drug resistance-associated mutations are persist at low levels by the time HIV-infected persons are diagnosed and genotyped. Using an approach that can more broadly identify variants, such as next-generation sequencing [42-45], may identify other mutations that would further increase the prevalence of drug resistance. However, because the more commonly transmitted mutations are often targeted by AS-PCR, any additional increase in mutation prevalence identified by the more complex methods may be nominal. Hence, the lower cost and simplicity of AS-PCR offer advantages for routine surveillance, particularly when the sample burden may be high. 

In conclusion, the relationship between minority drug resistance mutations and cART failure requires further exploration; nevertheless, the findings point to difficulties in getting infected persons diagnosed early and counseled to prevent forward spread of drug resistance. 

## Supporting Information

Table S1
**Oligonucleotide sequence Proportion(RTI mutations).**
(DOC)

Table S2
**Oligonucleotide sequence Proportion(PI mutations).**
(DOC)

Table S3
**Assay ΔCt measures, cutoffs and sensitivities on clinical samples.**
(DOC)
