# Supplementary material for: Highly-Sensitive Allele-Specific PCR Testing Identifies a Greater Prevalence of Transmitted HIV Drug Resistance in Japan
Source: PLoS One. 2013 Dec 16;8(12):e83150. doi: 10.1371/journal.pone.0083150 (PMC3865156; doi:10.1371/journal.pone.0083150)
Supplement: Table S1 — Oligonucleotide sequence Proportion(RTI mutations). (DOC) [file pone.0083150.s001.doc]

Table S1. Oligonucleotide sequence Proportion(RTI mutations)

|  | Primer | Oligonucleotide sequence | Proportion |
| --- | --- | --- | --- |
| Total copy reaction | ComFWD | 5'-CTT CTG GGA AGT TCA ATT AGG AAT ACC |  |
|  | ComREV | 5'-TGG TGT CTC ATT GTT TRT ACT AGG TA |  |
|  | Com 1P | 5'-***FAM***-TGG ATG TGG GTG A‘‘**T**’’G CAT ATT TYT CAR TTC CCT TA | 60% |
|  | Com 2P | 5'-***FAM***-TAC TGG ATG ‘‘**T**’’ GGG TGA TGC ATA TTT TTC ART TCC CTT A | 40% |
| Mutation |  |  |  |
| *Protease* |  |  |  |
| L90M | Rev1 | 5'-GAA AAT TTA AAG TGC AAC CAA KTT GAG TGA T |  |
|  | Fwd | 5'-AGA TCA CTC TTT GGC AAC GAC C |  |
|  | P1 | 5'-***FAM***-TAG GGG GAA ‘‘**T**’’TG GAG GTT TTR TCA AAG TAA GAC AGT AT |  |
|  |  |  |  |
| *Reverse transcriptase* |  |  |  |
| M41L | F1 | 5'-AAT AAA AGC ATT ART RGA AAT YTG TRC AGC AT | 35% |
|  | F2 | 5'-AAT WAA AGC ATT ART RGA AAT YTG TRC WGC AT | 10% |
|  | F3 | 5'-AAA AGC ATT ART RGA AAT YTG TRC AGG AC | 32% |
|  | F4 | 5'-TAA AAG CAT TAR TRG AAA TYT GTR CAK GTC | 13% |
|  | F5 | 5'-AAG CAT TAR TRG AAA TYT GTR CAK GGC | 10% |
|  | Rev | 5'-CCT AAT TGA ACT TCC CAG AAG TCT TG |  |
|  | 41-70.p | 5'-***FAM***-TTG GGC CTG AAA A‘‘**T**’’C CAT ACA ATA CTC CAG TAT TT |  |
|  |  |  |  |
| K65R | F1 | 5'- ACA ATA CTC CAR TAT TTG CCA TAA RCA G |  |
|  | Rev | 5'-CCT GGT GTC TCA TTG TTT ATA CTA GGT |  |
|  | K65R-P1 | 5'-***FAM***- TCA GAG AAC ‘‘**T**’’ TAA TAA RAG AAC TCA AGA CTT CTG GGA | 80% |
|  | K65R-P2 | 5'-***FAM***-TCA GAG AAC ‘‘**T**’’ CAA TAA GAG AAC TCA AGA CTT CTG GGA | 20% |
|  |  |  |  |
| K70R | Rev1 | 5'- GTT CTC TRA AAT CTA YTA WTT TTC TCC CTC | 70% |
|  | Rev2 | 5'-TTC TCT RAA ATC TAY TAW TTT TCT CCC CC | 30% |
|  | Fwd | 5'- AGA RAT TTG TAC AGA RAT GGA AAA GGA AG |  |
|  | 41-70.p | 5'-***FAM***-TTG GGC CTG AAA A‘‘**T**’’C CAT ACA ATA CTC CAG TAT TT |  |
|  |  |  |  |
| K103N | F1 | 5'-TCC HGC AGG GTT AAA RAA GGA C | 40% |
|  | F2 | 5'-ACA TCC MGC AGG GTT AAA AMA GGA T | 27% |
|  | F3 | 5'-CAT CCM GCA GGG TTA AAR VAG GAT | 11% |
|  | F4 | 5'-CAT CCI GCA GGI TTA AAA AAG GGC | 10% |
|  | F5 | 5'- T CCC KCW GGG TTA ARA AGG GAC | 12% |
|  | ComREV | 5'-TGG TGT CTC ATT GTT TRT ACT AGG TA |  |
|  | Com.3P | 5'-***FAM***-TGG ATG TGG GTG A‘‘**T**’’G CAT ATT TTT CAR TTC CCT TA |  |
|  |  |  |  |
| Y181C | F1 | 5'-AGR AAA CAA AAY CCA GAM ATA RTT GGC TG | 35% |
|  | F2 | 5'-ARA AAA CAA AAY CCA GAM ATA RTT GGA TG | 20% |
|  | F3 | 5'-AGR AAA CAA AAY CCA GAT MTA RTT GGC TG | 15% |
|  | F4 | 5'-ARA AAA AAA AAY CCA GAC MTA RTT GGC TG | 10% |
|  | F5 | 5'-AAA ACA AAA YCC AGA RAT ART CGG CTG | 10% |
|  | F6 | 5'-AAA ACA AAA YCC AGA RAT ART SGG CTG | 10% |
|  | 181-184.Rev | 5'-ATC AGG ATG GAG TTC ATA ACC CA |  |
|  | 181-184.P1 | 5'-***FAM***-TAG GAT CTG ACT TAG AAA ‘‘**T**’’ AGG RCA GCA TAG ARC | 80% |
|  | 181-184.P2 | 5'-***FAM***-TAG GAT CTG ATT ‘‘**T**’’ AGA AAT AGG RCA GCA TAG ARC | 20% |

Table S1. Cont.

| M184V | F1 | 5'-AAA TCC ARA MMT ART TAT MTR TCA GCA CG (ID No. 33) | 55% |
| --- | --- | --- | --- |
|  | F2 | 5'-AAA TCC ARA MAT AGW RAT MTR TCA GCA CG (NEW) | 25% |
|  | F3 | 5'-AAA YCC ARA MAT ART TAT CTR YCA GCA TG (ID No. 35) | 20% |
|  | 181-184.Rev | 5'-ATC AGG ATG GAG TTC ATA ACC CA |  |
|  | 181-184.P1 | 5'-***FAM***-TAG GAT CTG ACT TAG AAA ‘‘**T**’’ AGG RCA GCA TAG ARC | 80% |
|  | 181-184.P2 | 5'-***FAM***-TAG GAT CTG ATT ‘‘**T**’’ AGA AAT AGG RCA GCA TAG ARC | 20% |
|  |  |  |  |
| T215Y* | Rev1 | 5'-CTT TCT GAT GTT TYT KGT CTG GTG GAT | 20% |
|  | Rev2 | 5'-TTT CTG ATG TTT YTK GTC TGG TGC GT | 33% |
|  | Rev3 | 5'-TTT CTG ATR CTT TTY GTC TGG TGC GT | 22% |
|  | Rev4 | 5'-TTT CTG ATG TTT KTT GTC TGG GGC GT | 10% |
|  | Rev5 | 5'-TTT CTG ATG CTT TYT TTC TGG TGC GT | 15% |
|  | ComFwd | 5'-CTT CTG GGA AGT TCA ATT AGG AAT ACC |  |
|  | Com 1P | 5'-***FAM***-TGG ATG TGG GTG A‘‘**T**’’G CAT ATT TYT CAR TTC CCT TA | 60% |
|  | Com 2P | 5'-***FAM***-TAC TGG ATG ‘‘**T**’’ GGG TGA TGC ATA TTT TTC ART TCC CTT A | 40% |
|  |  |  |  |
| T215F# | Rev1 | 5'-TTT CTG ATG TTT YTG KTC TGG TGC GA | 50% |
|  | Rev2 | 5'-CTT TCT GAT GTT TYT GKT CTG GTG CAA | 50% |
|  | ComFwd | 5'-CTT CTG GGA AGT TCA ATT AGG AAT ACC |  |
|  | Com 1P | 5'-***FAM***-TGG ATG TGG GTG A‘‘**T**’’G CAT ATT TYT CAR TTC CCT TA | 60% |
|  | Com 2P | 5'-***FAM***-TAC TGG ATG ‘‘**T**’’ GGG TGA TGC ATA TTT TTC ART TCC CTT A | 40% |

FAM, 5-fluoro;

‘‘’’, nucleotide position where quencher is placed;

*includes intermediates 215D, H, and N;

#includes intermediates 215L, I and V.

$all sequences were published in reference 20.
