# Supplementary material for: Highly-Sensitive Allele-Specific PCR Testing Identifies a Greater Prevalence of Transmitted HIV Drug Resistance in Japan
Source: PLoS One. 2013 Dec 16;8(12):e83150. doi: 10.1371/journal.pone.0083150 (PMC3865156; doi:10.1371/journal.pone.0083150)
Supplement: Table S2 — Oligonucleotide sequence Proportion(PI mutations). (DOC) [file pone.0083150.s002.doc]

Table S2 Oligonucleotide sequence Proportion(PI mutations)

|  | Primer | Oligonucleotide sequence | Proportion |
| --- | --- | --- | --- |
| Mutation |  |  |  |
|  |  |  |  |
| L90M | L90M-R* | 5'-GAA AAT TTA AAG TGC AAC CAA KTT GAG TGA T |  |
|  | L90M-F* | 5'-AGA TCA CTC TTT GGC AAC GAC C |  |
|  | P1* | 5'-***FAM***-TAG GGG GAA ‘‘**T**’’TG GAG GTT TTR TCA AAG TAA GAC AGT AT |  |
|  |  |  |  |
| M46I | M46I-F | 5'-TGC CAG GAA RAT GGA RRC CAA AAC TA |  |
|  | M46I/L-R | 5'- GAA AAT TTA AAG TGC ARC CAA KCT GKG TCA |  |
|  | PRO2L** | 5'-TAT GGA TTT TCA GGC CCA ATT TTT GA |  |
|  | P1 | 5'-***FAM***-TAG GGG GAA ‘‘**T**’’TG GAG GTT TTR TCA AAG TAA GAC AGT AT |  |
|  |  |  |  |
| M46L | M46L-1F | 5'- TGC CAG GRA RAT GGA MAC CAA AGT | 50% |
|  | M46L-2F | 5'- TGC CAG GRA RAT GGA AGC CAA AGT | 30% |
|  | M46F-3F | 5'-TGC CAG GRA RAT GGA MAC CAA AGC | 20% |
|  | M46I/L-R | 5'- GAA AAT TTA AAG TGC ARC CAA KCT GKG TCA |  |
|  | PRO2L** |  |  |
|  | P1 | 5'-***FAM***-TAG GGG GAA ‘‘**T**’’TG GAG GTT TTR TCA AAG TAA GAC AGT AT |  |
|  |  |  |  |

FAM, 5-fluoro;

‘‘’’, nucleotide position where quencher is placed;

*all sequences were published in reference 20.

** for phylogenetic tree analysis
