# Supplementary material for: Highly-Sensitive Allele-Specific PCR Testing Identifies a Greater Prevalence of Transmitted HIV Drug Resistance in Japan
Source: PLoS One. 2013 Dec 16;8(12):e83150. doi: 10.1371/journal.pone.0083150 (PMC3865156; doi:10.1371/journal.pone.0083150)
Supplement: Table S3 — Assay ΔCt measures, cutoffs and sensitivities on clinical samples. (DOC) [file pone.0083150.s003.doc]

Table S3. Assay ΔCt measures, cutoffs and sensitivities on clinical samples.

|  | ΔCt cutoff | Cutoff mean % | Sensitivity, | Mean ΔCt (range) of wildtype sequences | Mean ΔCt (range) of | False-negatives |
| --- | --- | --- | --- | --- | --- | --- |
|  | (cycles) | mutant tested(%) | positive/mutants tested (%) | wildtype n = 42 | mutant samples | ΔCts |
|  |  |  |  |  |  |  |
| M46I | 11.0 | 0.54 | 55/55(100) | 18.67(16.39-26.65) | 4.34（1.39-10.1） | - |
|  |  |  |  |  |  |  |
| M46L | 9.0 | 4.01 | 22/22(100) | 18.98（15.43-23.43） | 5.26（0.88-8.95) | - |
|  |  |  |  |  |  |  |
